# Use of Handheld Video Otoscopy for the Diagnosis of Acute Otitis Media: Technical Note

**DOI:** 10.7759/cureus.5547

**Published:** 2019-09-01

**Authors:** Christy Soares, William Clifton, William D Freeman

**Affiliations:** 1 Neurology / Neurosurgery, Florida State University College of Medicine, Jacksonville, USA; 2 Neurosurgery, Mayo Clinic, Jacksonville, USA

**Keywords:** otoscope, technology, otitis media, smartphone, led, pediatrics, public health, diagnostic tools

## Abstract

Otoscopy is a simple, yet fundamental tool for medical practitioners of all levels to diagnose common otologic conditions. Otoscopy is traditionally performed by a handheld light with a lens. This technique has several disadvantages, especially during teaching sessions since only a first-person view is available. Video otoscopy has the ability to project the view of the scope onto a screen that can be displayed for medical or patient education. Recently, handheld video otoscopy has advanced to display compatibility with personal devices such as cell phones or tablets. In this technical report, we demonstrate components, setup, and use of video otoscopy for otologic examination that can be easily used on a personal electronic device.

## Introduction

Otitis media (OM) is an inflammation of the middle ear and can be subdivided into acute otitis media (AOM), chronic suppurative OM, and otitis media with effusion (OME). OM remains one of the most common causes of hearing loss worldwide and requires otoscopic examination for diagnosis [1]. Depending on the diagnosis, therapy indication changes. For example, OME usually does not require antibiotic therapy and instead watchful waiting, and antibiotics are only indicated if spontaneous recovery does not occur [2]. Similarly, AOM only requires antibiotic therapy when symptoms become severe [3]. Therefore, otoscopic examination training is vital to diagnosis and proper treatment. Video otoscopy is the use of otoscopic device that has a camera attached to the end that relays the image to a smart device. This technical note details the use of handheld video otoscope for this purpose.

## Technical report

**Figure 1:**
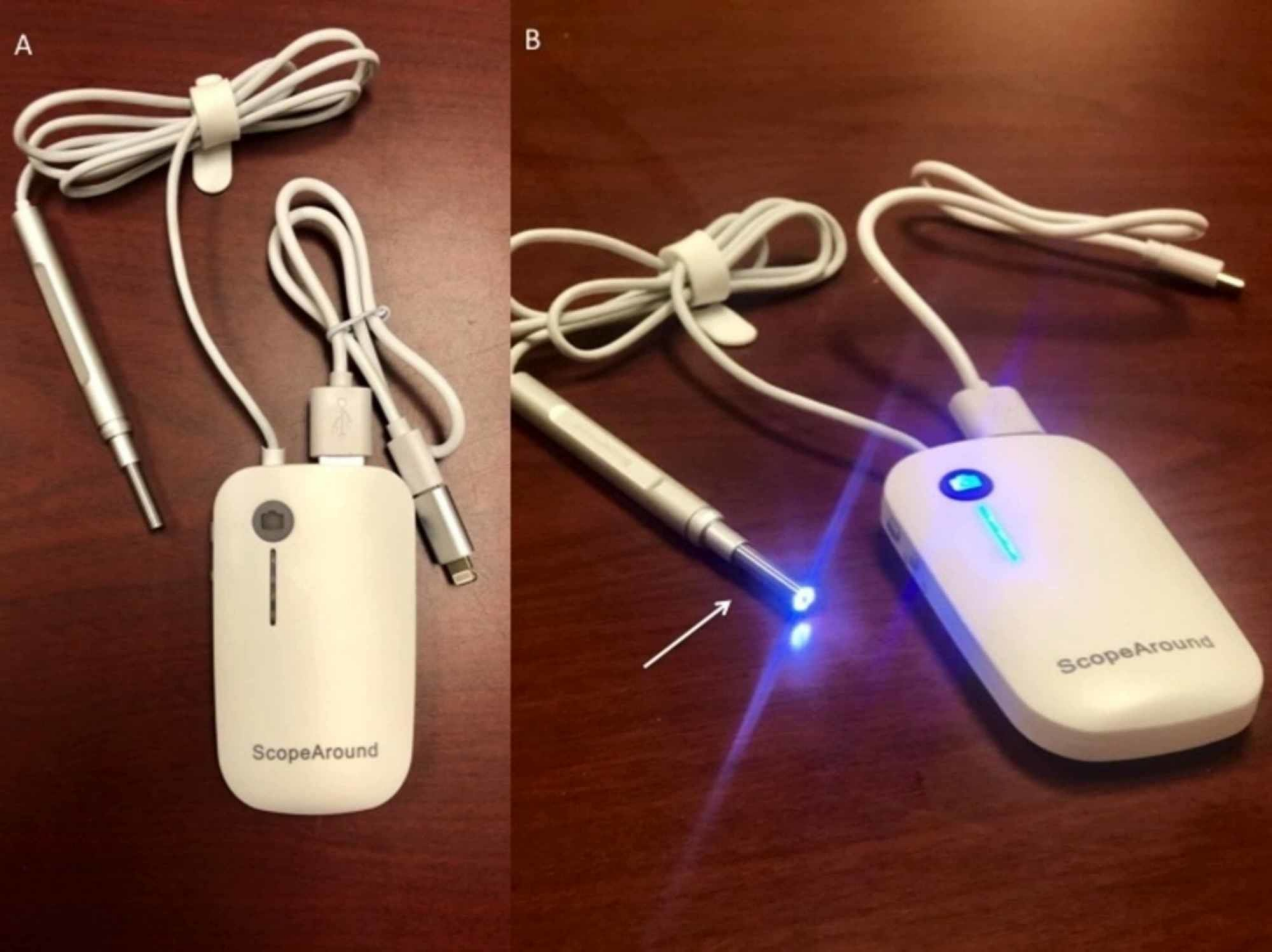
Video otoscope tool with attachable cord for smart device. (A) Component parts of the video otoscope. (B) The video otoscope has an LED light at the tip of the video recording device, with the ability to turn the light on and off without turning off the video recording (white arrow).

The video otoscope is easy to use and provides high-quality diagnostic evidence. It is relatively inexpensive ($25.99-$49.99) and is a handheld tool that plugs into any “smart” device. The video otoscope is about the size and width of a pencil with a camera at the end (see Figure 1). The video otoscope attaches via wire to the handheld device and produces the image from the camera.

**Figure 2:**
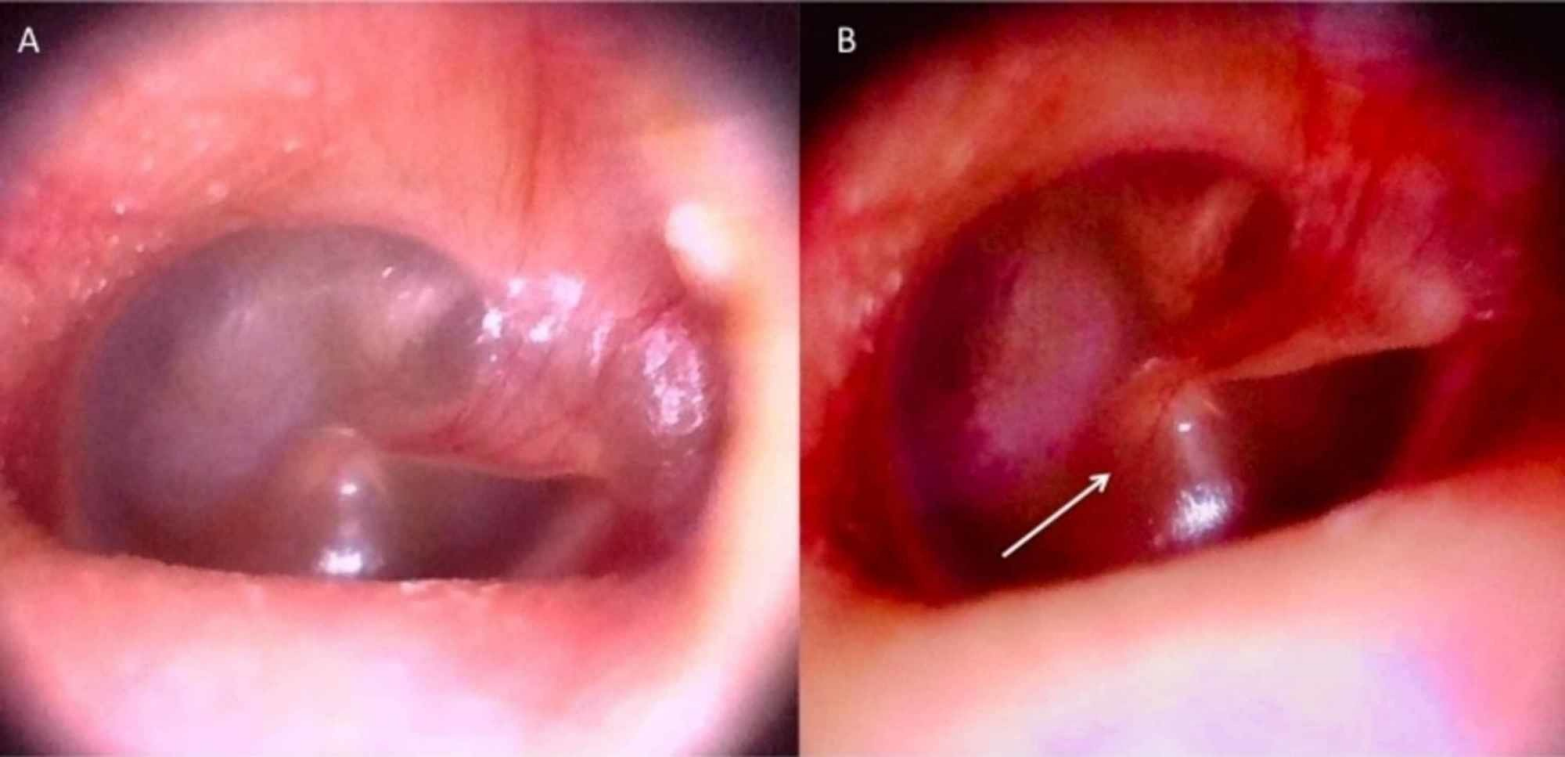
Stills from video otoscope (A) Image of normal tympanic membrane with clearly visible ossicles and relevant anatomy. (B) Image of otitis media with bulging tympanic membrane and diffuse erythema (white arrow)

For otoscopic examination, the otoscopic specula are then attached to the end of the camera in the same way they are attached to a normal otoscope. The camera is then inserted into the auditory canal for examination of the external auditory canal. The video otoscope also has attachable curettes. The curettes can be used to remove impacted wax and foreign objects from the auditory canal with a decreased risk of tympanic membrane perforation because the curettes are video guided. The otoscope also provides a clear view of the tympanic membrane and ossicles which may be recorded for medical record or research purposes (see Figure 2).

## Discussion

The potential scope of video otoscopy is vast, including as an educational tool, global health resource, and telehealth device. Video otoscopy can be a great educational resource for both clinicians and patients. Educationally, the ability to record or experience a patient’s examination with student and professor will improve clarity, provide immediate feedback, and provide a new avenue for teaching. Currently, medical students are not satisfied with their otoscopic training, with decreased confidence in diagnosing common conditions [4]. As a result, there is a need for improvement and standardization of how otoscopic examination is taught in medical schools across the country. With the use of video otoscopy, there is no need for medical students to guess if they indeed saw what their attending did. The use of video otoscopy phone applications allows the attending and the student to experience the examination simultaneously. With the ability to give real-time instruction and the ability to record the exam, ambiguity is removed from the experience. After the examination is over, the physician has the ability to review the exam as a video or as a still picture, with the capability to draw over the picture to identify structures and abnormalities in the patient that were examined. Similarly, this adds another layer of confidence with the ability of the attending to then ask specific questions of medical students to ensure comprehension. This technique is the epitome of the time old mantra, “See one. Do one. Teach one.” Lastly, video otoscopy was found to be equal or better than physical examination in diagnostic accuracy, especially in regard to grading of OM [3]. 

Global health efforts have their own unique barriers including limited resources, access to consultation and equipment, and, as seen in this case, barriers to effective communication. The intent of a global health effort is to bring the best medical care to those without access to that level of care, and a crucial aspect of healthcare access is availability of instrumentation. The video otoscopy provides an avenue for cerumen and foreign object removal, leading to improvement in hearing and balance. While this application is useful in otoscopic examinations, it can be expanded to nasal, eye, mouth, and even vaginal examinations. The light at the end of the camera could also be a useful tool in evaluating pupillary reflexes. With the ability to turn a light on or off and record the pupillary response in real time, the video can be replayed to establish the true response. For oral examinations, most video otoscopic devices are about the length of a tongue depressor but are more slender and have the potential to increase the visibility in the posterior oropharynx, for improved diagnostic capability. Similarly, if a clinician would like to consult another clinician, the recorded examination can be shared for consultation. The video recording is downloaded to the owner’s smart device without the use of Wi-Fi and after can be shared with colleagues. 

Lastly, it has implications in the booming field of telehealth. With the emergence of telehealth consultations and virtual appointments, the ability of the patient to have an in-home otoscope to record or live stream their own otological examination to share with the clinician for diagnosis is revolutionary. In-home video otoscopic devices will lead to more accurate diagnosis, leading to more effective treatment, and increased beneficial outcomes, thereby saving time, money, and energy for the patient and provider. 

## Conclusions

The use of video otoscopy is beneficial to clinicians, students, and patients. By utilizing readily available and cost-effective technology, education and skill of medical trainees can be maximized in a global setting.
